# Risk Factors and Clinical Outcomes of Recurrence in Adult Ovarian Granulosa Cell Tumors

**DOI:** 10.1002/cnr2.70036

**Published:** 2024-10-25

**Authors:** Mansoureh Golmohammadi Tavallaee, Malihe Hasanzadeh Mofrad, Zohreh Yousefi, Mansoureh Mottaghi, Fatemeh Homaei Shandiz, Behrouz Davachi, Bahram Hamidi, Marjaneh Farazestanian, Fahimeh Afzaljavan

**Affiliations:** ^1^ Department of Obstetrics and Gynecology School of Medicine, Mashhad University of Medical Sciences Mashhad Iran; ^2^ Supporting the Family and the Youth of Population Research Center Mashhad University of Medical Sciences Mashhad Iran; ^3^ Cancer Research Center Mashhad University of Medical Sciences Mashhad Iran; ^4^ Department of Radiology School of Medicine, Ghaem Hospital, Mashhad University of Medical Sciences Mashhad Iran; ^5^ Faculty of Medicine Mashhad University of Medical Sciences Mashhad Iran

**Keywords:** AGCT, granulosa cell tumors, prognosis, recurrence, risk factors

## Abstract

**Purpose:**

Granulosa cell tumors (GCTs) of the ovary are rare but clinically significant malignancies. Despite advances in treatment, recurrence has remained a substantial challenge. This study aimed to identify clinical outcomes and potential prognostic risk factors for recurrence in patients diagnosed with GCTs.

**Methods:**

In a retrospective cohort study, the ovarian cancer database of the gynecological tertiary referral cancer center, Mashhad University of Medical Sciences, Mashhad, Iran, was searched from August 2012 to August 2023 to find GCT cases. Demographic, clinical, pathological, intervention‐related factors, follow‐up, and survival findings were meticulously collected. Data were analyzed using SPSS v 23.

**Results:**

Ninety‐two patients with GCTs, including 86 AGCT and 6 JGCT subjects, were identified. Based on further analysis of AGCT patients, most patients were ages under 50 (58.1%), clinically presented pain (32.6%), and abnormal uterine bleeding (27.9%) as the most frequent symptoms. Stages IA (64.0%) and IC (20.9%) were common. Five‐year overall and progression‐free survival were 98.2% and 90.8%, respectively. With a median follow‐up time of 72 (0.0–180) months, disease recurrence was observed in 19 patients (23.9%), and five patients (5.4%) died of the disease. Stage IV was a hazard factor of recurrence (HR = 7.62, 95%CI (1.89–30.63); *p* = 0.004).

**Conclusions:**

The present study provides valuable insights into the outcomes and potential risk factors for recurrence in ovarian AGCTs. It duplicates the importance of stage in the prognosis of AGCT patients and highlights the safety of fertility‐sparing surgery in stage I and the lack of need to administer chemotherapy in stage IC.

## 1 Introduction

Granulosa cell tumors (GCT) are uncommon, nonepithelial neoplasms originating from the ovary. They are categorized into adult granulosa cell tumors (AGCT) and juvenile (JGCT) variants, collectively comprising less than 5% of all ovarian tumors and constituting 70% of ovarian sex cord‐stromal tumors. AGCT, the predominant subtype, affect primarily postmenopausal women aged 50–55, representing 95% of GCT cases. Conversely, JGCTs are considered borderline tumors prevalent in premenopausal individuals under 30 years of age [1]. While older studies provided a foundation for understanding GCTs, including the clinical presentation, histopathology, and hormonal aspects of GCTs, recent research has delved deeper into the molecular and genetic underpinnings of GCTs, leading to a more comprehensive understanding of tumorigenesis and potential therapeutic targets.

Despite their low incidence, GCTs possess significant clinical implications due to their potential for recurrence and associated symptomatology. Etiologically, AGCTs are linked to genetic aberrations in the FOXL2 gene, dysregulation of Sma and Mad‐related proteins, and the influence of transforming growth factor‐β. Additionally, promoter mutations within the TERT gene have been implicated in AGCT pathogenesis. In contrast, JGCTs exhibit AKT1 serine/threonine kinase activation as a primary oncogenic driver [2, 3, 4, 5, 6]. Epidemiological studies indicate a positive correlation between GCT risk and obesity, as well as a familial predisposition to breast or ovarian cancer. Conversely, oral contraceptives and multiparity appear to confer a protective effect against GCT development [7]. However, the relationship between AGCT incidence and parity or age at childbirth remains equivocal. Furthermore, postmenopausal hormone replacement therapy has not been demonstrated to increase the risk of subsequent AGCT [8].

Therapeutic interventions for GCTs are tailored based on age and disease stage. Surgical resection is the primary treatment modality for most patients, while chemotherapy, radiotherapy, and biological therapies are reserved for recurrent or metastatic disease. These adjunct treatments aim to prolong survival or extend disease‐free intervals. Post‐treatment surveillance involves regular clinical assessments, including medical history updates, pelvic examinations, and tumor marker evaluations. Suspicious findings necessitate diagnostic imaging, such as computed tomography. Given the early‐stage presentation of most GCTs, primary surgical intervention often results in curative outcomes. Unilateral salpingo‐oophorectomy (USO) is sufficient for pediatric and premenopausal patients due to the low incidence of bilateral disease at diagnosis. Conservative surgical approaches, including USO, omentectomy, and pelvic peritonectomy, have been reported as safe options for preserving fertility in women with advanced‐stage disease. Available evidence suggests that pregnancy does not adversely impact disease progression.

GCTs generally exhibit a favorable prognosis with a low malignant potential [9]. The tumor stage at diagnosis is the primary prognostic indicator, with advanced stages correlating with significantly reduced five‐year survival rates [10]. Long‐term outcomes demonstrate a decline in survival rates beyond the initial two decades, highlighting the chronic nature of the disease [11]. Recurrence is a common feature of GCTs, often occurring late and multiple times [12]. Several factors, including age (> 50 years), residual disease, elevated mitotic activity, tumor rupture, elevated CA‐125 levels (≥ 35 IU/mL), tumor size, and diabetes, are associated with increased recurrence risk and adverse prognosis [9, 13, 14, 15, 16, 17]. Fertility‐sparing surgical interventions in young women have demonstrated comparable reproductive outcomes and survival rates to radical surgical approaches, supporting the consideration of conservative management in appropriate cases [8]. While FOXL2 gene mutations and reduced expression of CD56, GATA‐4, and SMAD3 proteins have been linked to poorer prognoses, the prognostic significance of estrogen receptor, anti‐Mullerian hormone, and inhibin expression remains inconclusive. The prognostic value of mitotic rate, Ki‐67, p53, β‐catenin, and HER2 expression is inconsistent across studies [18].

Recurrent disease, particularly in AGCT, poses a substantial clinical challenge. Identifying factors predictive of recurrence is crucial for developing tailored treatment and surveillance strategies. While tumor stage and histopathological features may influence disease progression and outcome, the precise relationship between these variables and recurrence risk remains inadequately understood [19]. Despite advancements in the genetic and molecular characterization of GCTs, the rarity of the disease necessitates further research to establish robust population‐based epidemiological data, prognostic markers, and optimal treatment guidelines. To the best of our knowledge, there is no information on clinical management and outcome of Iranian AGCT patients; this study aimed to comprehensively characterize the baseline, clinical, and histopathological profiles of AGCT patients within a specific geographic region, while also evaluating the prognostic impact of chemotherapy regimens, and fertility‐sparing surgery surgical interventions on progression‐free and overall survival in a single‐center cohort study in the northeast of Iran.

## 2 Methods

### 2.1 Ethics

Under the ethical approval number IR.MUMS.IRH.REC.1402.042, this study was approved by the ethics committee of Mashhad University of Medical Science, Mashhad, Iran.

### 2.2 Study Population

A retrospective cohort study was conducted on a comprehensive ovarian cancer database of the gynecological tertiary referral cancer center at the educational and referral Qhaem Hospital, Mashhad University of Medical Sciences, Mashhad, Iran. Data from August 2012 to August 2023 were extracted to analyze the progression‐free survival in ovarian GCTs, considering clinic‐pathological characteristics.

The study population included individuals with a primary diagnosis of AGCT who underwent primary staging or surgery at any age in the educational and referral Qhaem Hospital by gynecological oncologist surgeons. The primary variables were meticulously selected to encompass a broad spectrum of factors potentially influencing GCT's recurrence and survival rates. These features included patient demographics, obstetric records, clinical evaluation, and comorbidities.

Age, BMI, number of pregnancies, live births, stillbirths, and abortions, infertility duration, and menopause were considered as demographics. Clinical presentation, including abnormal uterine bleeding, postmenopausal bleeding, masses, ascites, amenorrhea, signs of high androgens, pain, and the level of CA125 and Inhibin tumor markers, were evaluated.

The surgical intervention included uterine‐preserving USO and bilateral salpingo‐oophorectomy (BSO) plus total hysterectomy, and fertility‐sparing type of surgery. The surgery was performed by a gynecological oncologist. It was complete, with no residual lesions. Pathological features included tumor types (AGCT and JGCT), tumor location, tumor size, and the FIGO staging of the disease at the time of diagnosis. Moreover, endometrial thickness was recorded based on sonography reports. Considering the stage, the results of peritoneal cytology had been checked.

Prognosis‐related information, consisting of diagnosis date, the chemotherapy regimens administered to the patients, metastatic and recurrence events, follow‐up times, the date of therapy ended, and the living status of patients at the last follow‐up (alive and deceased), were recorded.

The data were measured and recorded using standardized medical records and laboratory tests. The staging of the disease was standardized according to the FIGO classification established in 2014 [20]. For patients who received treatment before 2014, their data were recalibrated to align with this classification.

### 2.3 Statistical Analysis

Statistical analyses were executed using SPSS software version 26 (Chicago, IL, USA). A *p* value of less than 0.05 was considered to indicate statistical significance.

The variables were analyzed using descriptive statistics, specifically mean (SD), median (IQR), or frequency (%) as appropriate. These variables were subsequently incorporated into the survival analysis to explore potential associations with recurrence survival.

The Kaplan–Meier method was implemented to estimate the progression‐free survival and compare it between different stages and pathology types using the Log Rank (Mantel‐Cox) test. To ascertain the hazard ratios for recurrence, a Cox regression analysis was conducted for baseline and clinical factors. The period of progression‐free survival was delineated as the interval commencing from the initial diagnosis to the occurrence of the first tumor recurrence, which mandated either medical or surgical intervention or resulted in death.

## 3 Results

### 3.1 Study Population Characteristics

A total of 92 GCT patients, including six JGCT and 86 AGCT cases, were included in the study. The mean ± SD age was 22.80 ± 6.91 and 44.89 ± 13.72 in JGCTs and AGCTs, respectively. As JGCT and AGCT have different ethologies, further analyses were conducted on AGCT subjects.

Table 1 indicates the demographic, clinical presentation, pathologic, and therapeutic characteristics of the study population. Of the 86 subjects of AGCT, 19 had recurrent disease. Also, metastasis was observed in 13 (15.1%) patients, including nine patients with involved lymph nodes and peritoneum and four patients with liver metastasis.

**TABLE 1:** Characteristics of the study population.

Variable	Overall (*N* = 86)	Recurrence	
		Positive (*N* = 19)	Negative (*N* = 67)
Age (years)	44.89 ± 13.72	46.26 ± 11.14	44.46 ± 14.42
< 50	50 (58.1%)	12 (63.2%)	38 (56.7%)
50–55	17 (19.8%)	2 (10.5%)	15 (22.4%)
56–60	9 (10.5%)	2 (10.5%)	7 (10.4%)
> 60	9 (10.5%)	3 (15.8%)	6 (9%)
BMI (kg/m^2^)	27.55 (22.0–47.75)	26.47 (23.01–46.99)	27.88 (22.0–47.75)
Number of pregnancies	3.00 (0.0–19.0)	3.0 (0.0–11.0)	3.0 (0.0–19.0)
Number of live births	2.00 (0.0–16.0)	2.5 (0.0–7.0)	2.00 (0.0–16.0)
Number of abortions	0.00 (0.0–3.0)	0.00 (0.0–3.0)	0.00 (0.0–3.0)
Number of stillbirth	0.00 (0.0–3.0)	0.00 (0.0–2.0)	0.00 (0.0–3.0)
Infertility duration (months)	13.50 (1.5–46.0)	16.5 (5.0–30)	12 (1.5–46.0)
Menopause	35 (40.7%)	7 (36.8%)	28 (42.4%)
Clinical presentation			
Abnormal uterine bleeding	24 (27.9%)	4 (21.1%)	20 (29.9%)
Postmenopausal bleeding	8 (9.3%)	1 (5.3%)	7 (10.4%)
Mass	9 (10.5%)	3 (15.8%)	6 (9.0%)
Ascites	4 (4.7%)	1 (5.3%)	3 (4.5%)
Amenorrhea	8 (9.3%)	1 (5.3%)	7 (10.4%)
Pain	28 (32.6%)	8 (42.1%)	20 (29.9%)
AUB and mass	1 (1.2%)	0 (0%)	1 (1.5%)
AUB and pain	1 (1.2%)	1 (5.3%)	0 (0%)
CA125 (U/ml)	24.5 (4.8–791.0)	23 (4.8–104.0)	24.75 (6.5–791.0)
Normal	33 (67.3%)	10 (76.9%)	23 (63.9%)
Abnormal	16 (32.7%)	3 (23.1%)	13 (36.1%)
Inhibin B (ng/mL)	15.20 (0.0–164)	69.5 (9.5–130)	8.6 (0.0–164.0)
Normal	19 (52.8%)	7 (87.5%)	12 (42.9%)
Abnormal	17 (47.2%)	1 (12.5%)	16 (57.1%)
Endometrial thickness (mm)	8.9 (1.0–41.0)	8 (1.0–41.0)	8.9 (3–29)
Pre‐menopause	8.5 (3.0–41.0)	7.0 (4.0–41.0)	8.7 (3.0–29.0)
Post‐menopause	9.5 (1.0–25.0)	12 (1.0–25.0)	9.15 (4.0–25.0)
Surgical characteristics			
USO (FSS)	30 (34.9%)	3 (15.8%)	27 (40.3%)
BSO + Total Hysterectomy	55 (64.0%)	16 (84.2%)	39 (58.2%)
Largest tumor diameter (mm)	96.5 (5.0–250.0)	54 (6–150)	100 (5.0–250)
Location of tumor			
Right ovary	38 (44.21%)	9 (47.4%)	29 (43.3%)
Left ovary	33 (38.4%)	6 (31.6%)	27 (40.3%)
Bilateral ovaries	9 (10.5%)	4 (21.1%)	5 (7.5%)
FIGO staging			
IA	55 (64.0%)	8 (42.1%)	49 (73.1%)
IC	18 (20.9%)	6 (31.6%)	13 (19.4%)
III	9 (10.5%)	2 (10.5%)	4 (6.0%)
IV	4 (4.7%) liver	3 (15.8%)	1 (1.5%)
Treatment & outcome			
Metastasis	13 (15.1%)	7 (36.8%)	6 (9.0%)
Chemotherapy	32 (37.2%)	14 (73.7%)	18 (26.9%)
Duration of follow‐up (months)	72 (0.0–180)	84 (12–180)	72 (0.0, 144)
Follow‐up status			
Alive	82 (95.3%)	18 (94.7%)	64 (95.5%)
Dead	4 (4.7%)	1 (5.3%)	3 (4.5%)

*Note:* Data are presented as Median (Min‐Max) or Frequency (%).

Abbreviations: BSO, bilateral salpingo‐oophorectomy; FSS, fertility‐sparing surgery; USO, unilateral salpingo‐oophorectomy.

The mean age was 44.89 ± 13.72 years, ranging between 13 and 84. Most patients were under 50 (58.1, *n* = 50), and 26 (30.3%) were between 50 and 59 years. The most number of pregnancies, live births, abortions, and stillbirths was 19, 16, 3, and 3, respectively. The median time of infertility was 13.50 (1.5–46.0); 26.7% of patients were in menopause, and 35 (40.7%) subjects were in menopause time.

Concerning clinical presentation, pain (32.6%) and abnormal uterine bleeding (27.9%) were more frequent than other factors, including postmenopausal bleeding, ascites, mass, and a combination of these signs and symptoms.

The level of tumor markers indicated a wide range, as CA125 (recorded for 49 patients) was between 4.8 and 791.0 with a median of 24.5 U/mL, and Inhibin B (registered for 36 patients) was between zero and 164 with a median of 15.20 ng/mL. Based on these tests, 16 (32.7%) patients had abnormal levels of CA125, and 17 (47.2%) had abnormal Inhibin B levels.

The surgical type included USO (or fertility‐sparing surgery, FSS) (*n* = 26, 34.9%) and (BSO) plus total hysterectomy (*n* = 55, 64.0%). The most frequent location of the tumor was in the right ovary (*n* = 38, 44.21%).

Based on FIGO staging, stages IA and IC were more common, as 57 (66.3%) patients were in IA and 19 (22.1%) were in IC. Stage II was not observed among subjects; the remaining was in stages III and IV. The recurrence rate was 14%, 32%, 33%, and 75% in stages IA, IC, III, and IV, respectively.

Chemotherapy was applied for 32 patients (37.2%), including seven (12%) in stage IA, 15 (79%) in stage IC, and all subjects of stages III (*n* = 6) and IV (*n* = 4). Among patients under chemotherapy, 4 (57.1%) in stages IA had recurrent disease after 71, 98, 160, and 178 months. Five (33.3%) patients in stages IC had a recurrence; two patients had a recurrence after 60 months, two patients after 72, and one patient after 96 months. Two patients in stage III (33.3%) had recurrence after 96 and 144 months, and three subjects in stage IV (75.0%) had recurrence after 62, 86, and 119 months.

### 3.2 Investigating Outcomes

**FIGURE 1:**
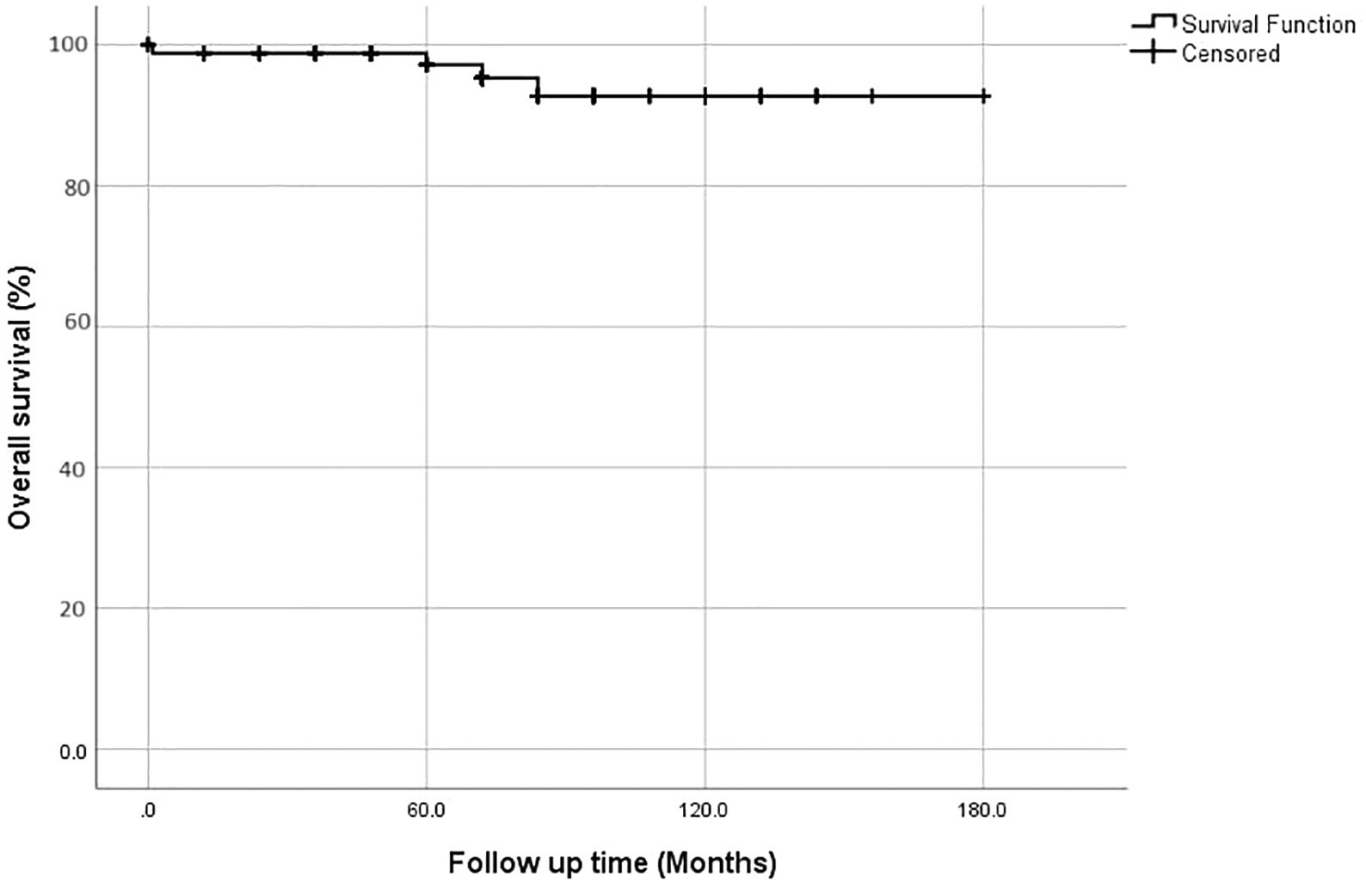
Overall survival of the study population. The estimated 5‐year overall survival was 98.2%.

**FIGURE 2:**
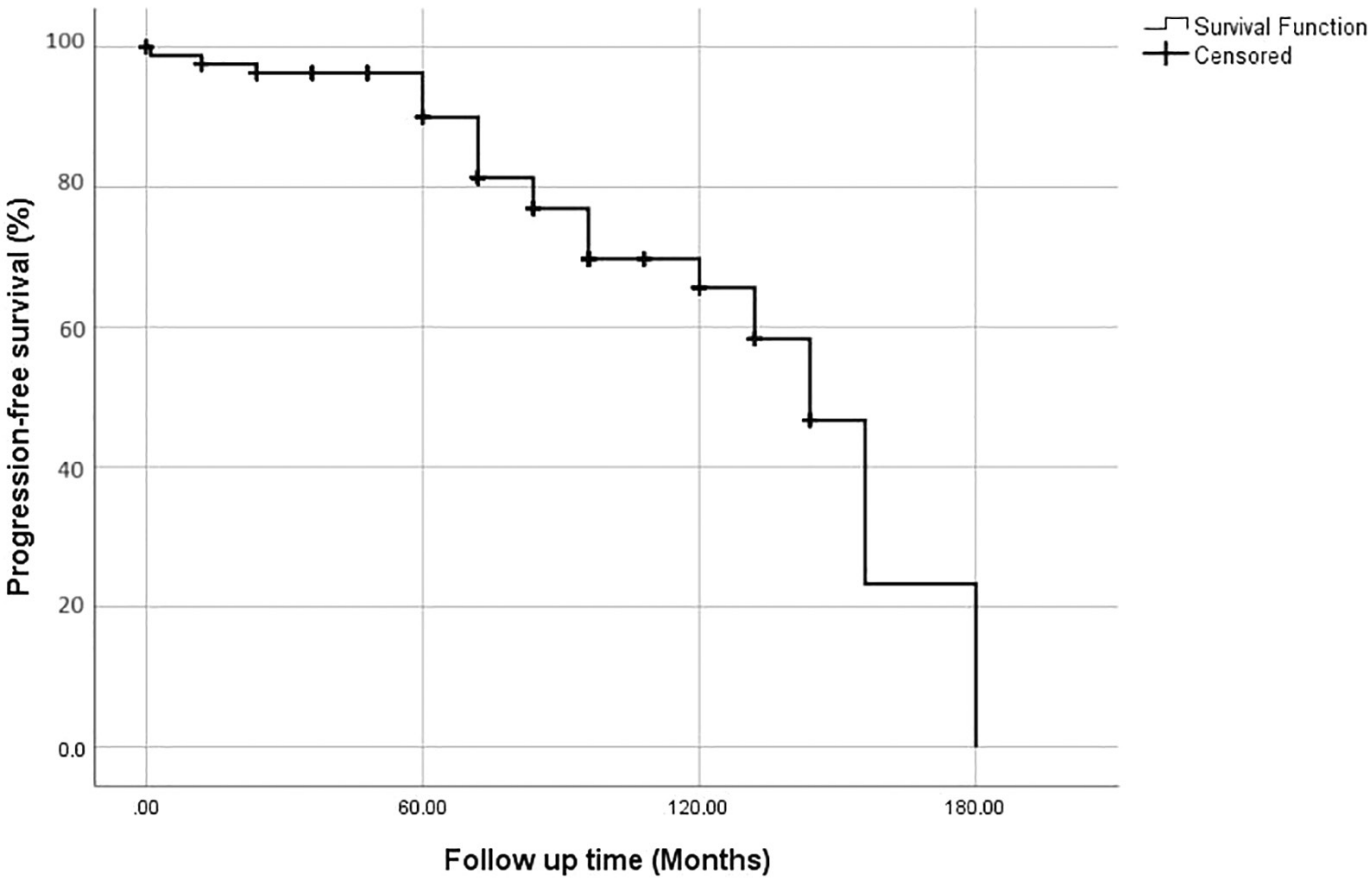
Progression‐free survival of the study population. The estimated 5‐year progression‐free survival was 90.8%.

The median follow‐up was 72 months (0–180). Nineteen patients (22.1%) had recurrent disease, and four patients (4.7%) died of the disease. The estimated 5‐year overall survival was 98.2% (Figure 1) and the 5‐year progression‐free survival was 90.8% (Figure 2).

### 3.3 Risk Factors for Recurrence

**FIGURE 3:**
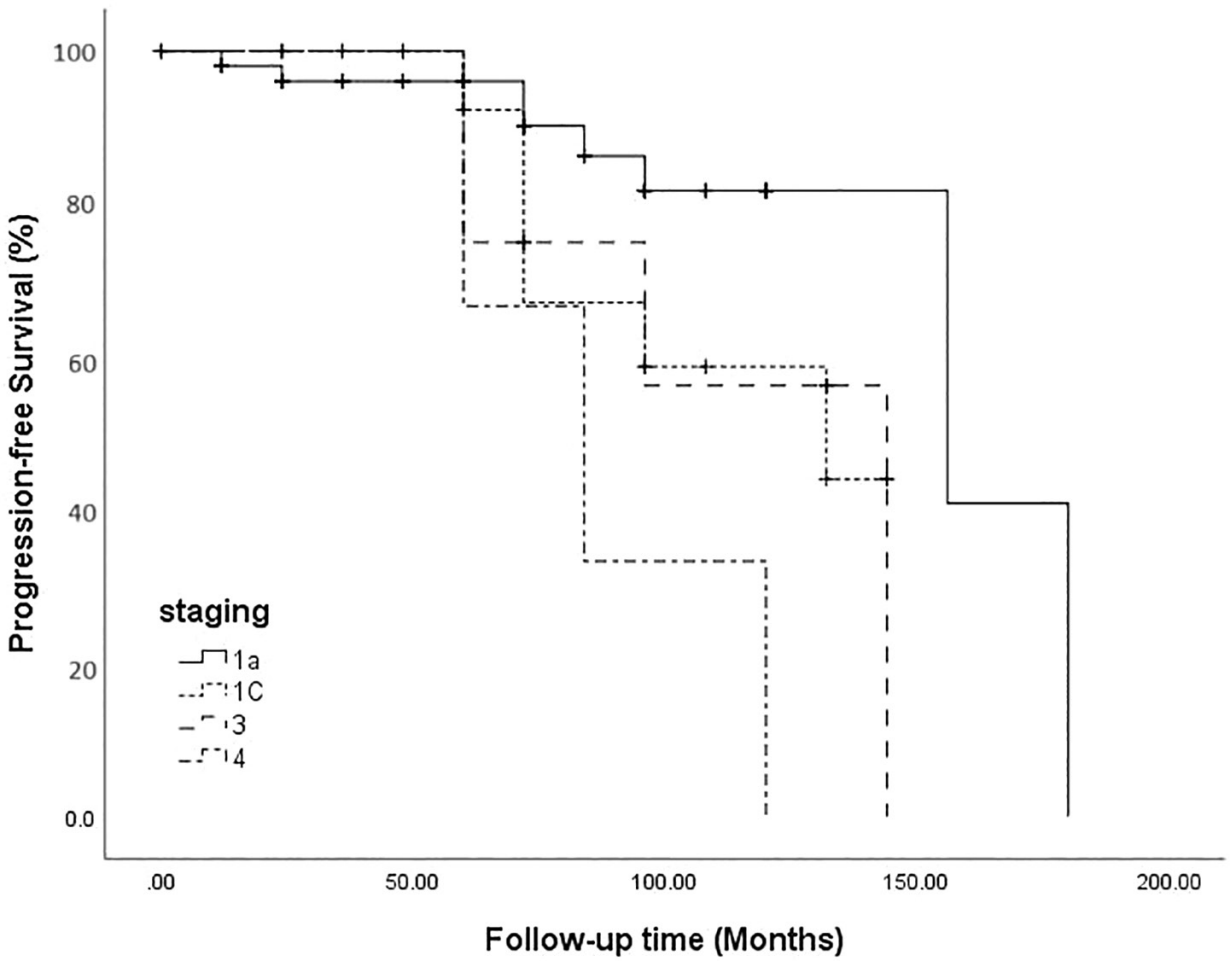
Progression‐free Survival based on Different Stages of Ovarian Granulosa Cell Tumors. The Log Rank (Mantel‐Cox) test indicated a statistically significant difference in recurrence survival distributions across stages IV and IA (*p* = 0.004).

Table 2 reports the univariate Cox regression analysis of considered potential hazard factors of prognosis in AGCT. Concerning demographics and biochemical markers, we did not find a hazard risk of prognosis. Moreover, surgery type, endometrial thickness, tumor location, tumor size, and chemotherapy did not affect the prognosis. The stage was only identified as a hazard factor. Stage IV disease was significantly associated with decreased progression‐free survival (HR = 7.62, 95% CI (1.89–30.63); *p* = 0.004) (Figure 3). Stage IV indicated significantly lower five‐year progression‐free survival than stage IA (*p* = 0.018). However, there was no significant difference between stages IV and IC.

**TABLE 2:** Risk factors for recurrence.

Characteristic	HR	95% CI		*p*
Age (Years)	0.99	0.96	1.04	0.918
50–55 versus < 50	0.61	0.17	2.19	0.454
56–60 versus < 50	0.81	0.18	3.64	0.781
> 60 versus < 50	0.93	0.26	3.40	0.916
BMI	1.06	0.96	1.16	0.238
Number of pregnancies	1.06	0.92	1.12	0.411
Number of live births	1.04	0.89	1.23	0.609
Number of abortions	1.64	0.82	3.26	0.161
Number of stillbirth	1.57	0.86	2.84	0.139
Infertility duration (Months)	1.01	0.91	1.12	0.856
Menopause (Yes vs. No)	0.94	0.38	2.30	0.892
CA125 level (U/ml) (Abnormal vs. Normal)	0.99	0.30	3.25	0.994
Inhibin B (ng/mL) (Abnormal vs. Normal)	0.16	0.02	1.25	0.081
Surgery (FSS vs. Non‐FSS)	1.60	0.45	5.68	0.469
Metastasis (Yes vs. No)	1.56	0.52	4.64	0.426
Endometrial thickness (mm)	1.01	0.94	1.08	0.890
Largest tumor diameter (mm)	1.00	0.98	1.01	0.426
Chemotherapy (Yes vs. No)	2.41	0.82	7.05	0.108
Stage IC versus IA	2.38	0.85	7.52	0.140
Stage III versus IA	2.88	0.80	10.38	0.107
Stage IV versus IA	7.62	1.89	30.63	**0.004**
Stage IA/surgery type (FSS vs. Non‐FSS)	0.58	0.11	2.99	0.513
Stage IC/surgery type (FSS vs. Non‐FSS)	0.04	0.00	123.52	0.422
Stage IA/Chemotherapy(Yes vs. No)	1.92	0.37	1.11	0.440
Stage IC/Chemotherapy (Yes vs. No)	0.68	0.08	6.11	0.732

*Note:* Significant *p*‐values are shown in bold.

Abbreviations: CI = confidence interval, HR = hazard ratio.

Considering stage IA and IC, administrating chemotherapy did not affect the recurrence (*p* > 0.05). Moreover, FSS was not associated with progression‐free survival in stages IA and IC (*p* > 0.05).

Univariate Cox regression indicated no significant difference between subjects with and without recurrence in all variables except the stage. However, adjusting for age, chemotherapy, and surgery type as the prominent markers of recurrent diseases revealed no change in the independent prognostic role of the stage.

## 4 Discussion

Our study provided a comprehensive analysis of the baseline characteristics, clinical outcomes, and potential recurrence risk factors in patients diagnosed with AGCTs of the ovary in Northeast Iran for the first time. The estimated 5‐year overall and progression‐free survival were 98.2% and 90.8% in a median follow‐up of 72 months (0–180). Our findings established a significant association between the FIGO stage and progression‐free survival, with stage IV tumors demonstrating a 7.9‐fold increased risk of recurrence compared to earlier stages. Notably, five‐year progression‐free survival differed between stage IV and IA but not stage IC. However, demographic, clinic‐pathological, and treatment variables did not influence the overall prognosis. FSS was deemed safe for stages IA and IC, while the efficacy of chemotherapy in improving progression‐free survival could not be assessed due to the universal administration of adjuvant chemotherapy in advanced‐stage (II‐IV) patients.

This investigation identified advanced‐stage disease (FIGO IV) as a critical prognostic factor associated with increased recurrence risk in patients with AGCTs. Notably, progression‐free survival in stage IC was comparable to that of stage III disease. Consistent with previous literature, our findings underscore the predominant role of disease stage in determining survival outcomes for ovarian GCTs [21, 22, 23]. Lee et al. found that the disease stage is the only factor associated with survival. Similarly, Ayhan et al. emphasized proper staging to determine disease progression, suggesting that the initial stage is the most crucial prognostic factor [22]. El Abbasi et al. also reported a stage‐based prognosis of the tumor [24]. Moreover, Plett et al. reported a comparable outcome of AGCT subjects with stage IC to those with advanced stages [21]. The significant association between the FIGO stage and recurrence underscores the imperative of accurate disease staging for tailoring personalized treatment plans. The presence of metastatic disease and the potential influence of chemotherapy on recurrence suggest the need for more intensive therapeutic approaches in specific patient subsets. These findings contribute to the evolving understanding of AGCT management and inform future research to optimize patient care.

The present study failed to demonstrate a statistically significant association between chemotherapy administration and recurrence in patients with AGCT. Furthermore, an unexpected finding was observed, with stage IC subjects receiving chemotherapy exhibiting lower five‐year PFS. The existing body of evidence offers limited support for the routine use of adjuvant chemotherapy in both early and advanced‐stage AGCT, underscoring the ongoing uncertainty surrounding its role in disease management. A recent study by Alhusaini et al. rejected the influence of chemotherapy on AGCT outcome [9]. A meta‐analysis reported modest response rates to chemotherapy, especially in subjects with stable disease [25], while a cohort study suggested potentially adverse outcomes [21]. Another meta‐analysis rejected the benefit of adjuvant chemotherapy, even for those with advanced or recurrent disease [26]. Due to the paucity of robust clinical data in this field, standardized chemotherapy guidelines for AGCT are currently unavailable. Treatment decisions should be individualized based on a comprehensive assessment of clinicopathologic presentation, underlying disease, fertility preservation considerations, and disease severity [27]. These findings highlighted the primacy of inherent tumor‐related factors, such as hemorrhage and tumor size, over treatment‐related factors. This perspective complements our study's focus on clinical and intervention‐related factors like metastasis, suggesting that a comprehensive risk assessment should incorporate both inherent and external factors.

Our study found that fertility‐sparing procedures did not significantly influence the recurrence rate, corroborating previous research by M. Ghalleb et al. [28]. Consistent with the literature, FSS has been established as a safe and effective treatment option for early‐stage malignant sex cord‐stromal tumors (MSCSTs) and germ cell tumors (GCTs) in women desiring to preserve fertility [29]. Moreover, FSS has not been shown to adversely impact DFS compared to radical surgery approaches, and pregnancy outcomes following FSS are generally favorable [30]. However, while FSS presents an attractive option for fertility preservation, the oncologic safety of this approach in premenopausal women remains a subject of ongoing debate [31]. Given the available evidence, comprehensive counseling regarding the potential need for subsequent surgical intervention is essential. For patients willing to commit to rigorous follow‐up, delaying radical surgery until recurrence may be considered an alternative approach [32, 33].

Given the propensity for late recurrences, previous investigations have emphasized the importance of long‐term follow‐up, given the late recurrence tendency of these tumors [22, 34]. While GCTs generally exhibit a favorable prognosis with a low malignant transformation rate, the prognostic significance of the disease stage at diagnosis is well‐established [35]. Patients with early‐stage disease (I) demonstrate excellent 5‐year disease‐specific survival rates, whereas survival outcomes progressively decline with the advancing stage at 41% and 98% for stages IV and I, respectively [36]. However, the long‐term prognosis is less optimistic, with substantial mortality rates observed beyond the initial two decades (a 20‐year survival rate of 66.8% and a global mortality rate of 30%–35%). Advanced age at diagnosis (over 50 years old) and residual lesions following surgical intervention have been identified as independent prognostic factors for adverse outcomes [11, 37]. Accordingly, extended follow‐up can be recommended, particularly in patients exhibiting risk factors for recurrence.

## 5 Limitations/Strength

This investigation represents the first single‐center, comprehensive analysis of Iranian patients with AGCT incorporating long‐term follow‐up data. Patient demographics and clinical information were derived from a regional oncology registry in Northeast Iran. The centralized nature of patient care within a single institution ensured consistent diagnostic and treatment protocols, minimizing interobserver variability and reducing potential biases related to diagnostic markers, surgical interventions, and therapeutic modalities. The substantial sample size of 86 AGCT cases provided a robust foundation for identifying epidemiological patterns and potential prognostic factors. However, the retrospective study design had inherent limitations, including potential selection bias. Additionally, the administration of chemotherapy in some stage IA patients introduced complexities in outcome interpretation due to a long‐lasting follow‐up study. Furthermore, the absence of comprehensive molecular profiling, including blood‐based markers and genetic mutations, represents a study limitation. The single‐center design may restrict the generalizability of the findings to broader populations.

## 6 Conclusions

The present study aligns with existing literature emphasizing the prognostic significance of the FIGO stage in AGCT patients. Moreover, our findings support the safety of fertility‐sparing surgical approaches and question the necessity of adjuvant chemotherapy for stage IC disease. These findings contribute to the growing body of knowledge regarding the epidemiology, clinical outcomes, and potential prognostic factors associated with ovarian AGCTs. The implications of this research are significant for managing this rare malignancy and provide a foundation for future studies aimed at refining treatment protocols. Given the limitations of this study, larger, multicenter, prospective investigations, and meta‐analysis approaches are warranted to validate the present findings and elucidate the underlying mechanisms influencing AGCT prognosis.

## Author Contributions

M.G.T., M.F., B.H., and F.A. conceived and designed the analysis. M.H.M., Z.Y., M.M., F.H.S., and B.D. collaborated in disease diagnosis and collecting the patients' data for the ovarian cancer database. M.G.T. and M.F. extracted the data. F.A. performed the analysis. M.G.T., M.F., and F.A. wrote the paper. All authors contributed to the final version of the manuscript.

## Ethics Statement

Under the ethical approval number IR.MUMS.IRH.REC.1402.042, this study was approved by the ethics committee of Mashhad University of Medical Science, Mashhad, Iran.

## Conflicts of Interest

The authors declare no conflicts of interest.

## Data Availability

The data that support the findings of this study are available from the corresponding author, [F.A. & M.F.], upon reasonable request.
